# Efficacy of a Self-Guided Internet Intervention With Optional On-Demand Feedback Versus Digital Psychoeducation on Sleep Hygiene for University Students With Insomnia: Randomized Controlled Trial

**DOI:** 10.2196/58024

**Published:** 2025-05-08

**Authors:** Anna-Carlotta Zarski, Karina Bernstein, Harald Baumeister, Dirk Lehr, Stella Wernicke, Ann-Marie Küchler, Fanny Kählke, Kai Spiegelhalder, David Daniel Ebert

**Affiliations:** 1 Division of eHealth in Clinical Psychology Department of Clinical Psychology Philipps University of Marburg Marburg Germany; 2 Psychology and Digital Mental Health Care TUM School of Medicine and Health Technical University Munich Munich Germany; 3 Department of Clinical Psychology and Psychotherapy Institute of Psychology and Education Ulm University Ulm Germany; 4 Department of Health Psychology and Applied Biological Psychology Leuphana University Lüneburg Lüneburg Germany; 5 Faculty of Applied Healthcare Sciences Deggendorf Institute of Technology Deggendorf Germany; 6 Department of Psychiatry and Psychotherapy Medical Center -- University of Freiburg, Faculty of Medicine University of Freiburg Freiburg Germany

**Keywords:** internet intervention, insomnia, university students, randomized controlled trial, active control group, cognitive behavioral therapy for insomnia, CBT-I

## Abstract

**Background:**

Internet-based cognitive behavioral therapy for insomnia (iCBT-I) provides flexibility but requires significant time and includes potentially challenging components such as sleep restriction therapy. This raises questions about its incremental effectiveness compared to less demanding minimal interventions such as sleep hygiene psychoeducation.

**Objective:**

This study aimed to assess the incremental efficacy of self-guided iCBT-I with optional on-demand feedback for university students with insomnia compared to a single session of digital psychoeducation on sleep hygiene.

**Methods:**

In a randomized controlled trial, 90 students with insomnia (Insomnia Severity Index ≥10) were randomly allocated to self-help–based iCBT-I (45/90, 50%) or one session of digital sleep hygiene psychoeducation with stimulus control instructions (active control group [aCG]: 45/90, 50%). The self-help–based iCBT-I consisted of 6 sessions on psychoeducation, sleep restriction, and stimulus control, including written feedback on demand from an eCoach. Assessments occurred at baseline (T1), 8 weeks after treatment (T2), and at a 6-month follow-up (T3) via web-based self-assessment and diagnostic telephone interviews. The primary outcome was insomnia severity at T2. Analyses of covariance were conducted in an intention-to-treat sample. Secondary outcomes included diagnoses of insomnia and major depression, sleep quality, sleep efficiency, worrying, recovery experiences, recovery activities, presenteeism, procrastination, cognitive irritation, and recuperation in sleep.

**Results:**

There was no difference in insomnia severity at T2 between the iCBT-I group (mean 11.27, SD 5.21) and aCG group (mean 12.36, SD 4.16; *F*_1,989.03_=1.12*; P*=.29; *d*=–0.26; 95% CI 0.68 to 0.17). A significant difference emerged at T3 (iCBT-I: mean 9.43, SD 5.36; aCG: mean 12.44, SD 5.39; *F*_1,426.15_=4.72; *P*=.03), favoring iCBT-I with a medium effect (*d*=–0.57; 95% CI 1.07 to –0.06). Most secondary outcomes revealed no significant differences between the groups. In total, 51% (23/45) of participants in the iCBT-I group completed all 6 sessions, and 69% (31/45) completed the 4 core sessions.

**Conclusions:**

In the short term, students might benefit from low-intensity, easily accessible digital sleep hygiene psychoeducation or iCBT-I. However, it appears that iCBT-I offers superiority over sleep hygiene psychoeducation in the long term.

**Trial Registration:**

German Clinical Trials Register DRKS00017737; https://drks.de/search/de/trial/DRKS00017737

## Introduction

### Background

University students commonly experience sleep deprivation, poor sleep quality, and disrupted sleep patterns due to irregular sleep-wake schedules influenced by extended study hours, social engagements, and noisy environments [1-4]. The lack of structured routines and the academic stress, personal development, financial concerns, and new social roles can exacerbate these sleep problems [5-7].

Among college students, the prevalence of insomnia is notably high, affecting up to half of the student population (range: 18.5%-30.5%) [8,9]. According to the *Diagnostic and Statistical Manual of Mental Disorders, Fifth Edition* (*DSM-5*), insomnia disorder is characterized by difficulties initiating and maintaining sleep. This involves frequent awakenings or difficulties resuming sleep, occurring at least 3 nights per week for a minimum of 3 months and causing notable distress or impairment in daily functioning [10].

Insomnia has far-reaching consequences, affecting health-related quality of life [11] and predicting the onset of depression, anxiety, alcohol misuse, and psychotic disorders [12]. Research suggests a link between inadequate sleep and elevated risk of self-harm and suicidal behaviors among university students [13]. Furthermore, insomnia is associated with diminished learning capacity as well as impaired neurocognitive functioning [14], placing students at risk of failing their academic goals [15-17]. In turn, good sleep quality has been identified as the foremost predictor of university students’ overall well-being [18].

Clinical guidelines in both Europe and the United States recommend cognitive behavioral therapy for insomnia (CBT-I) as first-line treatment [19-21]. CBT-I, a multicomponent intervention, addresses dysfunctional cognitions and behaviors contributing to sleep problems [22,23], including stimulus control, sleep restriction, sleep hygiene, relaxation techniques, and cognitive restructuring [24-28].

In the context of college students, CBT-I has yielded moderate to large improvements in sleep-related outcomes (standardized mean difference [SMD]=−0.55, *g*=0.61), and modest reductions in anxiety (SMD=−0.23) and depressive (SMD=−0.30) symptoms [29,30]. However, follow-up results have been mixed, ranging from significant medium effects to nonsignificant results [29,30], and studies using active comparison groups exhibit smaller effects on sleep outcomes [29].

Due to students’ digital proficiency and their inclination toward autonomous problem-solving, offering CBT-I via the internet (iCBT-I) appears to be a convenient and low-threshold alternative to traditional in-person CBT [31]. Overall, while CBT-I has demonstrated large effects on insomnia severity compared to a waitlist control (SMD=−1.27), both guided (SMD=−0.71) and unguided (SMD=−0.78) iCBT-I yielded medium effects compared to a waitlist-control condition [32]. Despite students’ high risk of insomnia, a recent meta-analysis identified only 4 iCBT-I randomized controlled trial (RCTs) in student samples with small to moderate effects (*d*=0.42) [29]. Among them, one small RCT in a sample of 61 undergraduate students was the sole study that compared iCBT-I to an active control group (aCG), which involved sleep education.

Moreover, the incremental efficacy of iCBT-I in comparison to active controls and minimal-dose interventions remains unclear, often due to the absence of practically sleep-relevant comparators in studies beyond treatment as usual, for example, puzzles [33]. Sleep hygiene psychoeducation makes for a suitable comparator, as it is part of clinical insomnia treatment [20] and commonly used in general practice [34]. Furthermore, psychoeducation offers a less intensive intervention in contrast to more demanding components such as sleep restriction, which could increase the likelihood of individuals discontinuing treatment. Stand-alone psychoeducational interventions focusing on sleep hygiene for college students have yielded moderate effects [35]. While they have been shown to be less effective than CBT-I [36], additional investigation is required to directly compare iCBT-I with sleep hygiene psychoeducation in student populations. Moreover, university students are a distinct demographic, with irregular work and sleeping schedules, high academic and social stress, loneliness, and frequent late-night digital device use—factors that exacerbate sleep disturbances [37]. These characteristics highlight the need for investigating tailored interventions in this group. Furthermore, guidelines emphasize the importance of thorough diagnostics, including clinical interviews, which currently lack in student-focused studies [20].

### Objectives

This study aimed to address these gaps by evaluating the incremental efficacy of self-guided iCBT-I with optional feedback on demand in comparison to an aCG that receives brief, low-intensity digital sleep hygiene psychoeducation in an RCT. We hypothesized the superiority of iCBT-I over the aCG in reducing insomnia severity at posttreatment and 6-month follow-up. A secondary objective was to exploratively investigate the impact of iCBT-I in comparison to aCG on additional sleep-related outcomes, such as observer-rated diagnoses of primary insomnia and major depression.

## Methods

### Study Design

This study was part of the German trial site (StudiCare) of the World Health Organization World Mental Health International College Student initiative [38,39]. A 2-armed RCT assessed the efficacy of student-specific iCBT-I versus digital sleep hygiene psychoeducation.

### Ethical Considerations

This study was approved by the ethics committee of the Friedrich-Alexander-Universität Erlangen-Nürnberg (481_18B) and registered in the German Clinical Trial Register DRKS00017737. The study adheres to the CONSORT (Consolidated Standards of Reporting Trials) [40] and the CONSORT-EHEALTH (Consolidated Standards of Reporting Trials of Electronic and Mobile Health Applications and Online Telehealth) criteria [41]. All participants gave written informed consent and were informed of their right to withdraw at any time without consequences. Data were collected pseudonymized using study IDs and are available in anonymized form after the final assessment and deletion of the coding list.

### Inclusion and Exclusion Criteria

We included university students who were (1) aged ≥18 years, (2) displaying heightened insomnia severity (Insomnia Severity Index [ISI] score ≥10 [42,43]), (3) German-speaking, (4) willing to give informed consent to participate in the study, and (5) had internet access. We excluded individuals who (1) reported that they had been diagnosed with a psychosis or bipolar disorder, (2) showed a notable suicidal risk (Beck Depression Inventory (BDI-II) item 9 score ≥1 [44]), and (3) were currently receiving or were on a waitlist for psychological treatment for any mental disorder.

### Study Procedures and Randomization

Participants from Germany, Austria, and Switzerland were recruited via (1) the StudiCare website, (2) social media and online forums, (3) mass emails sent through university mailing lists, and (4) distribution of flyers and posters in universities and public buildings. Participants with an ISI score ≥10 were invited to provide informed consent, complete the baseline self-report web-based assessment, and undergo a telephone-based clinical interview (approximately 15 minutes) to assess insomnia and major depressive disorder (MDD). Participants were then randomized to either iCBT-I group or aCG in block sizes of 4 and 6 by an individual not otherwise involved in the study, minimizing predictability while ensuring balanced allocation. Once randomized, participants received immediate access to either iCBT-I or 1 session of digital sleep hygiene psychoeducation.

### Intervention Group

The intervention was based on the iCBT-I GET.ON Recovery which incorporated established CBT-I treatment components such as sleep hygiene, stimulus control, sleep restriction, and cognitive interventions [45]. In addition, participants had the option to use activity and sleep diaries to support their progress. GET.ON Recovery has been shown to significantly reduce insomnia severity assessed with the ISI (*d*=1.45; 95% CI 1.06-1.84) in German teachers with work-related strain, compared to a waitlist-control group [45,46]. The intervention has also demonstrated efficacy in various formats compared to a waitlist: unguided among employees (*d*=1.37, 95% CI 0.99-1.77) [47], with adherence-focused guidance, including reminders and feedback on demand (*d*=1.63; 95% CI 1.23-2.03) for severe insomnia in a working population [48], and in a 3-session version without sleep restriction (g=0.84; 95% CI 0.39-1.30) for international university students [49]. In this study, the intervention was specifically tailored to the needs of the university population. The adapted intervention consisted of 6 sessions and participants were instructed to complete 1 session per week. It was tailored specifically to students based on prior analyses identifying their treatment needs for internet-based interventions [50]. Considering the link between stress, poor sleep quality, and insomnia in college students [51], additional treatment components on recreational activities to reduce hyperarousal and enhance cognitive detachment and restorative sleep were added. The tailored intervention addressed common sleep-related issues reported by students, including irregular routines, study-related distractions, academic stress, and limited leisure time. Moreover, students’ requests for comprehensive, evidence-based information influenced the adaptation of the iCBT-I to better suit their needs [50].

The first session provided information about recovery and sleep hygiene, explaining the connections between sleep, cognitive hyperarousal, cognitive detachment, and leisure activities. Participants were introduced to a 10-item web-based recovery diary to monitor sleep efficiency, including time in bed and total sleep time. The second session focused on stimulus control and sleep restriction techniques, guiding participants to reschedule their sleep habits in the ensuing weeks. In the third session, participants reviewed their sleep restriction process, making adjustments based on sleep efficiency. This session also introduced recovery-promoting behaviors based on behavioral activation [52]. The fourth session combined cognitive interventions with psychoeducation to address negative repetitive thinking associated with sleep-inhibiting hyperarousal. The fifth session incorporated elements from metacognitive therapy [53], teaching techniques to reduce continuous attention to negative repetitive thinking regarding both sleep and study-related stress. Each session included transfer tasks for integrating recreational activities, monitoring sleep efficiency, and implementing sleep hygiene and restriction into daily life. The sixth session focused on relapse prevention, preparing participants for potential decreases in sleep quality during high-stress periods, and offering strategies for stress management and coping. The intervention was self-help-based with the option for participants to request semistandardized feedback on completed sessions from a trained bachelor-level psychology student on demand [54], although the use of this feedback option was minimal. Adherence reminders were automatically sent by the eHealth platform system when a session remained unfinished for over 7 days.

### Active Control Group

Participants in the aCG received 1 session on sleep hygiene psychoeducation through the same web-based platform used by the participants in the iCBT-I group. Digital psychoeducation was comprised of (1) sleep education about causes and maintaining factors of insomnia and (2) sleep hygiene information, including techniques such as stimulus control. The aCG did not include any exercises and behavioral activities or interactive material. After 6 months, aCG participants were offered access to iCBT-I.

### Measures

Outcomes, unless otherwise specified, were assessed at baseline before randomization (T1), posttreatment 8 weeks after randomization (T2) and 6-month follow-up (T3) via self-report web-based assessments on the platform LimeSurvey and observer-rated structured clinical interviews via telephone by blinded trained psychologists.

### Primary Outcome: Insomnia Severity

Insomnia severity was assessed with the German version of the ISI (7 items, score range: 0-28; Cronbach α=0.83) [42,43,55]. The ISI evaluated the nature, severity, and impact of insomnia over the last month, considering aspects such as sleep onset and maintenance difficulties, early morning awakening, sleep dissatisfaction, interference with daily functioning, noticeability by others, and distress. Higher scores indicated more severe insomnia symptoms. A web-based measure of the ISI has been validated [56]. Total scores allowed classification into the following severity levels: absence (0-7), subthreshold (8-14), moderate (15-21), and severe insomnia (22-28) [43].

### Secondary Outcomes

#### Insomnia

A diagnosis of insomnia was assessed via the Structured Clinical Interview for Sleep Disorders based on the *DSM-5* criteria [57]. The criteria require symptoms of difficulty initiating or maintaining sleep, or early morning awakenings, occurring at least 3 nights per week for a minimum of 3 months. These symptoms must result in significant distress or functional impairment and cannot be explained by another sleep-wake disorder, substance use, medical condition, or mental disorder.

#### Depression

The presence of current MDD according to *DSM-5* was assessed via the Structured Clinical Interview for DSM-5–Clinical Version [58]. A diagnosis required the presence of 5 or more symptoms over a 2-week period, including at least one core symptom: either depressed mood or loss of interest or pleasure. Additional symptoms included significant weight changes, sleep disturbances, psychomotor agitation or retardation, loss of energy, feelings of worthlessness or excessive guilt, impaired concentration, or recurrent thoughts of death or suicide. These symptoms must cause clinically significant distress or impairment in social, occupational, or other important areas of functioning and must not be attributable to the physiological effects of a substance or another medical condition.

#### Sleep Quality

Sleep quality was measured with item 6 of the Pittsburgh Sleep Quality Index (PSQI; score range: 0-3; “How would you overall describe your sleep quality within the last four weeks?”) [59]. A lower score represented better sleep quality.

#### Sleep Efficiency

Sleep efficiency was measured with items 1, 3, and 4 of the PSQI. Participants were asked to record their evening bedtimes, their rising times, and their total hours of sleep within the past 4 weeks [60]. Sleep efficiency was then computed using the following formula: total hours of sleep/time spent in bed. Good sleep efficiency is indicated by ≥85% [61]. Higher scores reflected better sleep efficiency.

#### Worrying

We measured worrying as one form of hyperarousal in the past week with the short version of the 3-item Penn State Worry Questionnaire (score range: 0-18; α=0.87) [62,63] using the German version [64]. Higher scores reflected stronger worrying.

#### Recovery Experiences

Recovery experiences were measured with the 16-item Recovery Experience Questionnaire (score range: 16-80) [65] assessing psychological detachment, which involves relaxation (4 items; α=0.68), mastery (4 items; α=0.86), control (4 items; α=0.82), and the absence of hyperarousal (4 items; α=0.89). Higher scores reflected greater recovery experiences.

#### Recovery Activities

The 21-item Recreation Experience and Activity Questionnaire (score range: 21-105; α=0.72) assessed participants’ frequency of recreational activities during the past week (0, 1, 2, 3, or ≥4 times) [66]. Greater scores indicated increased engagement in recovery activities.

#### Presenteeism

The work impairment score of the presenteeism scale for students was used to measure reduced academic performance due to health problems within the past 4 weeks; for example, “Were you tired because you lost sleep?” (10 items; score range: 20-100; α=0.81) [67]. Higher scores indicated a lower level of presenteeism.

#### Procrastination

To measure procrastination, we used the 7-item procrastination scale for students (score range: 7-35; α=0.94) [68], where participants specified the extent to which they postponed study tasks over the past 2 weeks. Higher items indicated more pronounced procrastination behavior.

#### Cognitive Irritation

Work-related hyperarousal was assessed with the cognitive irritation subscale of the 3-item Irritation Scale (score range: 3-21; α=0.87) [69]. Higher scores indicate heightened hyperarousal.

#### Recuperation in Sleep

To measure recuperation in sleep, the respective subscale of the 8-item sleep questionnaire was used (score range: 8-40; α=0.81) [70]. Higher scores represented better recuperation in sleep.

#### User Satisfaction

User satisfaction was assessed at T2 with the 8-item Client Satisfaction Questionnaire adapted to the web-based intervention context (score range: 8-32; α=0.93); elevated scores align with greater satisfaction levels, with scores >23 denoting substantial satisfaction [71,72].

### Sample Size

To calculate the required sample size, we used an effect size for global measures of sleep disturbances of *g*=0.79 (95% CI 0.52-1.06) derived from a meta-analysis on psychological interventions to improve sleep in college students [30]. According to the sample size calculation using G*Power, a sample of 90 participants was necessary to detect a large effect (*d*=0.79) of iCBT-I compared to an aCG concerning the primary outcome, with a power (1−β) of 80% and an α level of 0.05 (2-tailed test).

### Statistical Analysis

Our analyses followed CONSORT guidelines [41,73] and were based on the intention-to-treat (ITT) principle. Missing data in both ITT and intervention completer analyses were handled under the missing at random assumption. We generated 50 imputation sets using fully conditional specification and multivariate imputation by chained equations [74], with all variables set as predictors. The imputed results were synthesized following Rubin’s rules [75]. Sensitivity analyses were performed, encompassing study completer and intervention completer analyses (Multimedia Appendix 1). Study completer analyses included participants who completed the primary outcome assessment at T2 or T3, respectively, while intervention completer analyses included those who finished at least 4 out of 6 modules. All analyses were conducted using R statistical software (version 4.0.4; R Foundation for Statistical Computing) [76] and RStudio (version 1.4.1106) [77]. Reported *P* values were 2-sided with a significance level of.05.

For the primary outcome at T2 and T3, we used an ITT-based univariate analysis of covariance (ANCOVA) with baseline ISI scores as covariates, comparing iCBT-I to aCG [78,79]. ANCOVAs were also used to explore secondary outcomes. Between-group effect sizes were reported as Cohen *d* with 95% CIs. At T2, treatment response was evaluated through reliable change and symptom-free status in the primary outcome. Reliable change was determined using the reliable change index [80], indicating a decrease of >1.96 (equivalent to 5.01 points on ISI) from T1 to T2 and T1 to T3. Symptom-free status was defined as an ISI score ≤8 at T2 and T3 [43,46]. The treatment response was compared between iCBT-I and aCG using Pearson chi-square test. To evaluate diagnosis-free status in insomnia and MDD, we included participants who met the diagnostic criteria for these disorders at baseline. We calculated the number of participants who maintained or transitioned to a diagnosis-free status at T2 and T3. We compared diagnosis-free status between iCBT-I and aCG groups by conducting logistic regressions of insomnia diagnosis, while adjusting for baseline insomnia severity. We calculated the number needed to treat (NNT) and its corresponding 95% CI indicating the number of participants that must be treated to achieve one additional treatment response [81,82]. Intervention adherence was measured based on the average number of completed overall sessions and the number of participants who completed all 4 core sessions.

## Results

### Participant Characteristics and Descriptive Data

**Figure 1:**
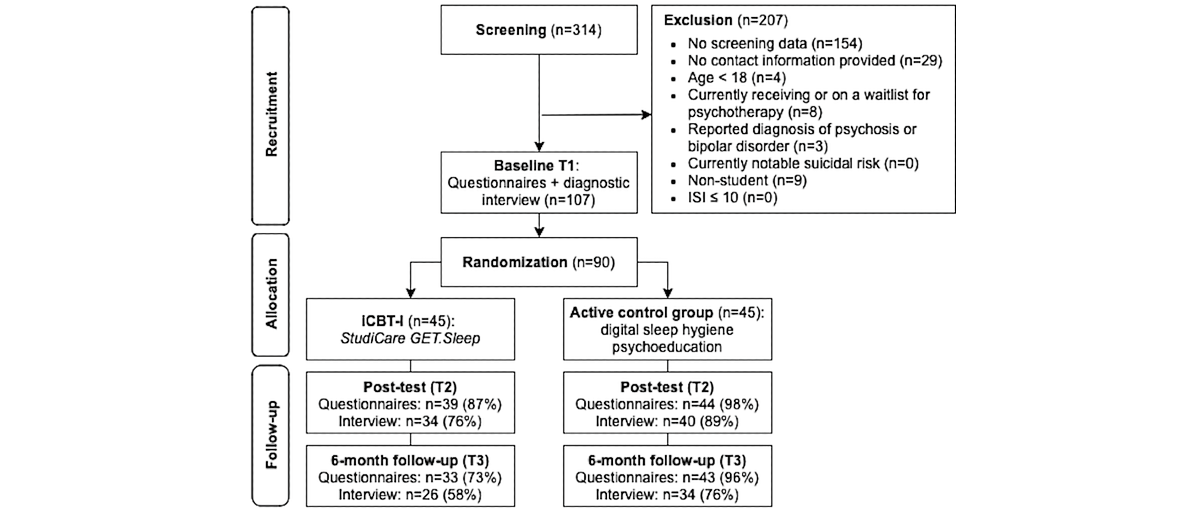
Study flow. iCBT: intervention group; iCBT-I: internet-based cognitive behavioral therapy for insomnia; ISI: Insomnia Severity Index.

The study took place from August 2019 to May 2021. After screening (n=160), 53 applicants were excluded, mainly due to lack of informed consent (n=29; Figure 1). A total of 90 participants were randomized to iCBT-I group (45/90, 50%) or aCG (45/90, 50%). Baseline self-report and clinical interview data were available for all participants. The web-based assessment was completed by 92% (83/90) of the participants at T2 and 84% (76/90) at T3, and the clinical telephone interview by 82% (74/90) of the participants at T2 and 67% (60/90) at T3. The participants were on average aged 24.97 (SD 4.51) years, predominantly women (64/90, 71%), and either in a relationship or married (46/90, 51%), as depicted in Table 1. The most cited reason for using an internet-based intervention was the inclination to manage insomnia independently (83/90, 92%), followed by perceiving internet-based interventions as an attractive treatment option (38/90, 42%). At baseline, iCBT-I group and aCG did not differ in insomnia diagnosis (iCBT-I: 43/45, 95% and aCG: 43/45, 95%). Descriptive ITT data are depicted in Table 2. Descriptive study and intervention completer data can be found in Multimedia Appendix 1.

**Table 1:** Sociodemographic characteristics for the intention-to-treat sample.

Characteristics			Total (N=90)		iCBT-I^a,b^ (n=45)		aCG^c^ (n=45)
Age (y), mean (SD)			24.97 (4.51)		24.86 (4.1)		25.07 (4.93)
Gender (women), n (%)			64 (71)		32 (71)		32 (71)
Married or in a relationship, n (%)			46 (51)		25 (56)		21 (47)
Country of residence (Germany); n (%)			85 (94)		42 (93)		43 (96)
Immigration background, n (%)			19 (21)		8 (18)		11 (24)
German as native language, n (%)			88 (98)		45 (100)		43 (96)
Children, yes; n (%)			2 (2)		1 (2)		1 (2)
Level of education, high; n (%)			90 (100)		45 (100)		45 (100)
**Academic degree, n (%)**							
	None	39 (43)		22 (49)		17 (38)	
	Bachelors	36 (40)		18 (40)		18 (40)	
	Masters or diploma	15 (17)		5 (11)		10 (22)	
**Planned academic degree, n (%)**							
	Bachelors	23 (26)		13 (29)		10 (22)	
	Masters	40 (44)		18 (40)		22 (49)	
	State examination	20 (22)		12 (27)		8 (18)	
	Diploma	2 (2)		1 (2)		1 (2)	
	Other	5 (6)		1 (2)		4 (9)	
Working, n (%)			51 (57)		21 (47)		30 (67)
**Financial situation, n (%)**							
	No financial issues	80 (89)		43 (96)		37 (82)	
	Financial issues	10 (11)		2 (4)		8 (18)	
Experience with internet-based health programs, n (%)			9 (10)		3 (7)		6 (13)
**Motivation to participate in an internet-based intervention, n (%)**							
	Preference for self-help	83 (92)		41 (91)		42 (93)	
	Interested in internet-based interventions	38 (42)		10 (22)		28 (62)	
	Fear of stigmatization with in-person psychotherapy	11 (12)		8 (18)		3 (7)	
	No other contact point found	9 (10)		5 (11)		4 (9)	
	Not being able to specify the problem	9 (10)		3 (7)		6 (13)	
	Embarrassment with in-person psychotherapy	9 (10)		6 (13)		3 (7)	
	Waiting times for psychotherapy too long	5 (6)		3 (7)		2 (4)	
	Prior treatment not helpful	3 (3)		0 (0)		3 (7)	
	No access to psychotherapy on site	3 (3)		3 (7)		0 (0)	

^a^iCBT-I: Internet-based cognitive behavioral therapy for insomnia.

^b^Intervention group.

^c^Active control group.

**Table 2:** Means and SDs of the internet-based cognitive behavioral therapy for insomnia (iCBT-I) intervention group and the active control group (aCG) at T1, T2, and T3 for the intention-to-treat sample (N=90).

Outcome			T1^a^, mean (SD)					T2^b^, mean (SD)					T3^c^, mean (SD)		
			iCBT-I^d^		aCG		iCBT-I			aCG		iCBT-I			aCG
**Primary outcome**															
	Insomnia severity^e^	16.51 (36.5)		16.29 (3.09)		11.27 (5.21)			12.36 (4.16)		9.43 (5.36)			12.44 (5.39)	
**Secondary outcomes**															
	Sleep quality (PSQI^f^)^g^	1.00 (0.48)		1.04 (0.52)		1.52 (0.72)			1.40 (0.49)		2.24 (0.66)			2.36 (0.57)	
	Sleep efficiency (PSQI)^h^	73.10 (15.28)		75.04 (12.94)		81.80 (14.34)			78.34 (11.96)		83.86 (11.07)			80.51 (12.87)	
	Cognitive irritation (IS^i^)^j^	13.47 (4.77)		12.33 (5.24)		12.39 (5.08)			11.01 (5.23)		11.53 (5.51)			11.88 (5.51)	
	Worrying (PSWQ^k^)^l^	9.04 (3.92)		9.51 (4.16)		7.95 (3.97)			8.20 (3.71)		7.82 (4.28)			8.05 (4.20)	
	Recovery experiences (REQ^m^)^n^	48.31 (6.90)		49.93 (8.12)		49.32 (9.03)			50.89 (8.29)		52.04 (10.05)			52.73 (9.48)	
	REQ_psychological detachment	10.62 (3.26)		11.71 (3.31)		11.29 (3.45)			11.97 (3.47)		12.61 (3.86)			12.45 (4.08)	
	REQ_relaxation	12.71 (2.71)		12.18 (2.83)		13.01 (2.84)			13.16 (2.71)		13.18 (3.40)			13.22 (3.60)	
	REQ_mastery	10.31 (2.20)		11.20 (3.75)		10.92 (3.66)			10.80 (3.95)		10.98 (3.46)			11.96 (3.49)	
	REQ_control	14.67 (3.03)		14.84 (2.66)		14.18 (3.35)			14.95 (2.44)		15.38 (3.65)			15.06 (2.60)	
	Recovery activities (ReaQ^o^)^p^	30.02 (10.06)		28.11 (10.06)		30.52 (11.55)			32.19 (12.30)		31.18 (9.53)			29.26 (11.11)	
	Recuperation in sleep (SFB^q^)^r^	17.82 (4.91)		17.73 (4.17)		20.51 (6.01)			19.29 (5.67)		21.63 (6.24)			19.82 (5.02)	
	Presenteeism (PSS^s^ Subscale WIS^t^)^u^	46.22 (15.70)		48.78 (13.33)		42.77 (16.05)			43.40 (14.46)		46.30 (18.95)			43.52 (13.64)	
	Procrastination (PFS^v^)^w^	21.58 (8.13)		21.13 (7.07)		19.37 (7.36)			20.61 (6.88)		21.00 (8.58)			21.09 (6.75)	

^a^T1=baseline before randomization.

^b^T2=8-week posttreatment.

^c^T3=6-month follow-up.

^d^Intervention group.

^e^Insomnia Severity Index (0-28), with higher scores reflecting higher insomnia symptoms.

^f^PSQI: Pittsburgh Sleep Quality Index.

^g^Sleep quality (0-3), with lower scores reflecting better sleep quality.

^h^Sleep efficiency (0-100), with higher scores indicating increased sleep efficiency.

^i^IS: Irritation Scale.

^j^Irritation Scale (3-21), with higher scores indicating heightened hyperarousal.

^k^Penn State Worry Questionnaire.

^l^Penn State Worry Questionnaire (0-18), with higher scores reflecting stronger worrying.

^m^REQ: Recovery Experience Questionnaire.

^n^Recovery Experience Questionnaire (16-80), with higher scores reflecting better recovery experiences, subscales psychological, detachment, relaxation, mastery, control (4-20) with higher scores reflecting better recovery experiences.

^o^ReaQ: Recreation Experience and Activity Questionnaire.

^p^Recreation Experience and Activity Questionnaire (21-105), with higher scores indicating increased engagement in recovery activities.

^q^German sleep questionnaire.

^r^German sleep questionnaire (8-40), with higher items reflecting higher recuperation in sleep.

^s^PSS: Presenteeism Scale for Students.

^t^WIS: Work improvement score.

^u^Presenteeism scale for students, work impairment score (20-100), with higher scores reflecting a lower level of presenteeism.

^v^PFS: Procrastination scale for students.

^w^Procrastination scale for students (7-35), with higher scores indicating higher procrastination behavior.

### Primary Outcome Analysis

Participants in the iCBT-I group did not show significantly lower ISI scores compared to the aCG at T2 (iCBT-I: mean 11.27, SD 5.21; aCG: mean 12.36, SD 4.16; *F*_1,989.03_=1.12*; P*=.29). At T3, insomnia symptom severity was significantly lower in the iCBT-I group than in the aCG group (iCBT-I: mean 9.43, SD 5.36; mean 12.44, SD 5.39; *F*_1,426.15_=4.72; *P*=.03) with a moderate effect (*d*=−0.57; 95% CI 1.07-0.06). The ITT ANCOVA results are depicted in Table 3.

**Table 3:** Between-group effects at T2 and T3 for the intention-to-treat sample (N=90).

Outcome		Between-groups effect at T2^a^	ANCOVA^b^		Between-groups effect at T3^c^	ANCOVA	
		*d* (95% CI)	*F* test (*df*)	*P* value	*d* (95% CI)	*F* test (*df*)	*P* value
**Primary outcome**							
	Insomnia severity (ISI^d^)^e^	−0.26 (0.68 to 0.17)	1.12 (1, 989.03)	.29	−0.57 (–1.07 to −0.06)	4.72 (1, 426.15)	.03
**Secondary outcomes**							
	Sleep quality (PSQI^f^)^g^	0.21 (–0.23 to 0.64)	1.33 (1, 2604.65)	.41	–0.17 (−0.61 to 0.28)	0.72 (1, 1286.36)	.40
	Sleep efficiency (PSQI)^h^	0.27 (–0.10 to 0.64)	0.95 (1, 661.38)	.33	0.33 (–0.14 to 0.79)	1.45 (1, 678.88)	.23
	Cognitive irritation (IS^i^)^j^	0.15 (–0.24 to 0.53)	1.85 (1, 2121.05)	.17	–0.14 (−0.62 to 0.34)	0.38 (1, 3925.22)	.54
	Worrying (PSWQ^k^)^l^	−0.04 (−0.49 to 0.41)	0.14 (1, 2304.26)	.71	−0.03 (−0.48 to 0.43)	0.21 (1, 6798.13)	.65
	Recovery experiences (REQ^m^)^n^	−0.06 (−0.46 to 0.34)	0.803 (1, 977.91)	.37	0.04 (−0.38 to 0.45)	0.23 (1, 3474.75)	.63
	REQ_psychological detachment	−0.03 (−0.44 to 0.39)	0.92 (1, 1253.52)	.34	0.16 (−0.33 to 0.65)	0.28 (1, 1981.98)	.59
	REQ_relaxation	−0.13 (−0.56 to 0.30)	0.176 (1, 4603.47)	.68	−0.10 (−0.54 to 0.34)	0.25 (1, 2820.68)	.62
	REQ_mastery	0.17 (−0.22 to 0.56)	0.168 (1, 6696.85)	.68	−0.15 (−0.59 to 0.29)	1.62 (1, 509.34)	.20
	REQ_control	−0.22 (−0.62 to 0.17)	1.65 (1, 453.31)	.20	0.13 (−0.27 to 0.53)	0.31 (1, 2545.75)	.57
	Recovery activities (ReaQ^o^)^p^	−0.22 (−0.62 to 0.18)	0.47 (1, 3772.46)	.49	0.10 (−0.32 to 0.52)	0.75 (1, 1229.44)	.39
	Recuperation in sleep (SFB^q^)^r^	0.20 (−0.16 to 0.55)	1.34 (1, 3837.72)	.25	0.31 (−0.13 to 0.02)	2.03 (1, 1045.50)	.15
	Procrastination (PFS^s^)^t^	−0.22 (−0.54 to 0.10)	1.30 (1, 656.62)	.25	−0.04 (−0.45 to 0.37)	0.27 (1, 6944.05)	.60
	Presenteeism (PSS^u^)^v^	0.06 (−0.34 to 0.47)	0.28 (1, 2838.04)	.60	0.24 (−0.23 to 0.70)	0.65 (1, 851.02)	.42

^a^T2=8-week posttreatment.

^b^ANCOVA: analysis of covariance.

^c^T3=6-month follow-up.

^d^ISI: Insomnia Severity Index.

^e^Insomnia Severity Index (0-28), with higher scores reflecting higher insomnia symptoms.

^f^PSQI: Pittsburgh Sleep Quality Index.

^g^Sleep quality (0-3), with lower scores reflecting better sleep quality.

^h^Sleep efficiency (0-100), with higher scores indicating increased sleep efficiency.

^i^IS: Irritation Scale.

^j^Irritation Scale (3-21), with higher scores indicating heightened hyperarousal.

^k^Penn State Worry Questionnaire.

^l^Penn State Worry Questionnaire (0-18), with higher scores reflecting stronger worrying.

^m^Recovery Experience Questionnaire.

^n^Recovery Experience Questionnaire (16-80), with higher scores reflecting better recovery experiences, subscales psychological, detachment, relaxation, mastery, control (4-20) with higher scores reflecting better recovery experiences.

^o^ReaQ: Recreation Experience and Activity Questionnaire.

^p^Recreation Experience and Activity Questionnaire (21-105), with higher scores indicating increased engagement in recovery activities.

^q^SFB: German sleep questionnaire.

^r^German sleep questionnaire (8-40), with higher items reflecting higher recuperation in sleep.

^s^PFS: procrastination scale for students.

^t^Procrastination scale for students (7-35), with higher scores indicating. higher procrastination behavior.

^u^PSS: Presenteeism Scale for Students.

^v^Presenteeism scale for students, work impairment score (20-100) with higher scores reflecting a lower level of presenteeism.

### Secondary Outcome Analyses

No statistically significant differences in secondary outcomes were found between iCBT-I and aCG in the ITT analyses at both time points (Table 3). iCBT-I and aCG did not differ in insomnia diagnosis at T2 (iCBT-I: 21/43, 49%; aCG: 27/43, 63%; χ^2^=-0.6; *P*=.18; NNT=7.36; 95% CI −5.91 to 2.86) or T3 (iCBT-I: 16/43, 37%; aCG: 24/43, 56%; χ^2^=–0.86; *P*=.16; NNT=5.02, 95% CI 2.59-2.9; in Multimedia Appendix 1). At T1, none of the iCBT-I participants met the criteria for MDD (0/45, 0%), while only 1 participant in the aCG (1/45, 2%) did. At T2, 1 iCBT-I participant was diagnosed with MDD (1/34, 3%), whereas none in the aCG met the criteria (0/40, 0%). At T3, 1 aCG participant met the criteria for MDD (1/34, 3%), and none in the iCBT-I did (0/26, 3%; Table S8 in Multimedia Appendix 1).

### Treatment Response

According to the reliable change index, 11% (5/45) of iCBT-I participants and 16% (7/45) of aCG participants demonstrated reliable improvement in insomnia severity at T2 (χ^2^=0.07; *P*=.76; NNT=−22.50; 95% CI −5.41 to 10.45). Reliable change remained constant at T3 for aCG (7/45, 16%), while more iCBT-I participants showed reliable improvement in insomnia severity at T3 (16/45, 35.6%; χ^2^=3.7; *P*=.05; NNT=5.00; 95% CI 2.66-40.70). At T2, 20.0% (9/45) of iCBT-I participants were symptom-free, compared with 13% (6/45) in the aCG (χ^2^=0.3; *P*=.57; NNT=15.00; 95% CI −11.53 to 4.54). At T3, iCBT-I had significantly more symptom-free participants (19/45, 42%) compared to aCG (7/45, 16%) at T3 (χ^2^=6.5; *P*=.01; NNT=3.75; 95% CI 2.24-11.41).

### Intervention Completer Analyses

In the intervention completer analysis at T2 (Multimedia Appendix 1), participants who completed the iCBT-I had significantly lower insomnia severity compared to those in the aCG (iCBT-I: mean 10.00, SD 4.25; aCG: mean 12.36, SD 4.16; *F*_1,4245.70_=5.73; *P*=.02; *d*=−0.49; 95% CI −0.95 to 0.02). This between-group difference was also present at T3 (iCBT-I: mean 9.00, SD 4.90; aCG: mean 12.44, SD 5.39; *F*_1,1795.80_=6.92; *P*=.009; *d*=−0.61; 95% CI −1.10 to −0.13). Regarding secondary outcomes at T2, the iCBT resulted in significantly higher sleep efficiency (iCBT-I: mean 83.44, SD 11.17; aCG: mean 78.34, SD 11.96; *F*_1,935.50_=4.33; *P*=.04; *d*=0.41; 95% CI 0.01-0.81), greater recuperation in sleep (iCBT-I: mean 22.21, SD 5.28; aCG: mean 19.29, SD 5.67; *F*_1,3575.13_=6.78; *P*=.009; *d*=−0.32; 95% CI −0.08 to 0.73), and lower procrastination levels (iCBT-I: mean 40.74, SD 15.58; aCG: mean 43.40, SD 14.46; *F*_1,2001.49_=5.75; *P*=.02; *d*=−0.23; 95% CI −0.52 to 0.06) compared to the aCG. However, at T3, mastery regarding the recovery experience was significantly lower in the iCBT-I group compared to the aCG (iCBT-I: mean 10.48, SD 3.24; aCG: mean 11.96, SD 3.49; *F*_1,2400.53_=4.83; *P*=.03; *d*=−0.29; 95% CI −0.69 to 0.12). No significant differences were observed in other secondary outcomes at both time points (Multimedia Appendix 1).

### Treatment Adherence and Satisfaction With the Intervention

Overall, 51% (23/45) of iCBT-I participants completed all 6 sessions, while 69% (31/45) finished the 4 core sessions. Specifically, 87% (39/45) of the participants completed session 1, 82% (37/45) session 2, 73% (33/45) session 3, 69% (31/45) session 4, 60% (27/45) session 5, and 51% (23/45) session 6. On average, participants went through 4.22 (70%) sessions of the intervention. In the iCBT-I group, user satisfaction was mediocre (mean 16.66, SD 4.69), with 79% (30/38) of participants willing to recommend the intervention to a friend in need. In the aCG, user satisfaction was significantly higher (mean 22.23, SD 4.32; t_80_=5.59; *P*<.001).

## Discussion

### Principal Findings

The results of this study indicate that self-help-based iCBT-I and digital sleep hygiene psychoeducation result in similar short-term improvements in university students with insomnia after 8 weeks, with no significant differences between the 2 groups. However, over the long term, after 6 months, iCBT-I demonstrated superior efficacy, showing medium-sized effects (*d*=−0.57), greater reliable change improvements, and significantly higher rates of symptom-free status. No significant between-group effects were found for any of the secondary outcomes. If the recommended minimal dosage of 4 sessions was completed, iCBT-I was observed to be more effective than digital sleep hygiene psychoeducation also in the short term and with respect to secondary outcomes.

### Comparison With Prior Work

The between-group effects observed in this study are considerably smaller compared to the effects in other iCBT-Is, that is, the original GET.ON Recovery intervention [46,47,83], which, however, has been evaluated in comparison to waitlist-control groups. A recent study also revealed reduced effects of iCBT-I when compared to active as opposed to waitlist-control conditions [29]. The within-group changes in insomnia severity for the iCBT-I group in this study were notably smaller compared to trials evaluating the original version of the intervention in both guided and unguided formats. This suggests that, beyond differences stemming from the choice of comparator group, the intervention may be less effective in this sample of university students. Lower baseline insomnia severity scores, indicating less impairment, may have limited the potential for improvement, contributing to the smaller observed changes. Nevertheless, it is noteworthy that the baseline-to-6-month improvement of approximately 7 points in this trial is still close to the −8.4-point change score typically associated with moderate improvement after treatment [43]. Another reason for the smaller between-group effects at posttreatment, compared to earlier trials in other samples, might be the use of the German ISI version in this study, which references a 4-week period. This longer reference period may have been less sensitive to detecting potential changes at posttreatment, whereas earlier studies used a 2-week reference period.

The finding of lower efficacy in this sample compared to other populations aligns with meta-analytic evidence indicating that digital interventions tend to be less effective in younger populations across various mental health outcomes [84]. Notably, a stress-management intervention with the same study design showed smaller effects in college students receiving feedback on demand compared to an older employee sample receiving content-focused feedback, with the latter showing almost double the effect in the longer term (*d*=0.57 vs *d*=1.07) [85,86]. This age-related and target group-specific effect is also evident in this study, which demonstrates similar effects to other iCBT-Is in college students at posttreatment (*d*=0.42) [29] and follow-up (*g*=0.56) [30]. One explanation could be that older participants with higher levels of self-discipline may better engage in self-help interventions, completing modules and exercises that transfer to daily life.

The study results suggest that digital sleep hygiene psychoeducation might be sufficient as an initial treatment step in stepped care for some students with insomnia to reduce its severity in the short term. On average, ISI scores decreased by 3.93 points in the digital sleep hygiene psychoeducation group, an effect that nearly reaches the threshold for a slight practically meaningful effect (4.7 points) as defined by Morin et al [43]. One possible explanation for the relatively large effects of the aCG intervention could be that sleeping problems among university students might be primarily driven by poor sleep hygiene rather than complex psychophysiological processes. Therefore, these issues might be effectively addressed through sleep hygiene psychoeducation alone, with limited additional benefits from incorporating other core components of iCBT, such as sleep restriction therapy. This assumption is supported by recent findings from a pilot trial with international students [49], which demonstrated significant baseline-to-follow-up improvements in insomnia severity using a significantly shortened version of the intervention (3 instead of 6 sessions) that excluded sleep restriction therapy. Moreover, the aCG intervention included not only psychoeducation on sleep hygiene but also stimulus control instructions, an element typically absent from such interventions. Stimulus control therapy has been identified as a key effective component of CBT-I [87] and is recommended as a stand-alone treatment for chronic insomnia in clinical guidelines [21]. Its inclusion in both intervention conditions may have contributed to the limited differences observed between groups. However, it is important to note that sleep hygiene and stimulus control instructions were provided only as informational content, without interactive delivery within the module or corresponding transfer tasks. In addition, the well-educated participants showed a strong inclination to independently address their sleep problems, potentially enabling them to initiate behavioral changes solely through digital sleep hygiene psychoeducation.

The results may also indicate that expectancy and attention effects, or spontaneous remission, might be primarily responsible for the observed effect [88]. In addition, the study’s contextual elements, including human interaction during diagnostic interviews, might have played a role in enhancing outcomes [89]. The observed significant primary effect at 6 months could be attributed to iCBT-I providing additional strategies for implementing and sustaining behavioral changes, including coping with recurring sleep problems during stressful examination phases, surpassing the benefits of digital sleep hygiene psychoeducation alone. Alternatively, students in the iCBT-I condition may have required more time to engage with the detailed content and practice exercises to fully realize its effects. The absence of significant secondary outcomes contradicts the small to moderate positive meta-analytic effects for associated sleep- and mental health–related outcomes in iCBT-I [90,91].

Furthermore, the satisfaction rate in the iCBT-I group was lower compared to the aCG and the original GET.ON Recovery intervention with close contact to a health care professional [46]. Expectation effects may have influenced treatment satisfaction, as iCBT-I participants may have anticipated greater contact and support from an eCoach in a CBT intervention compared to the level provided in iCBT-I with feedback on demand. However, satisfaction rates were also notably lower in this study compared to a trial evaluating the original intervention in an unguided format (mean 26.98, SD 5.12) [47]. A potential explanation for the lower satisfaction could be the demanding nature of iCBT-I, which requires significant participant effort due to its multisession structure. In addition, treatment components such as sleep restriction may be perceived as less enjoyable, further contributing to reduced satisfaction. Moreover, meeting students’ desire for more intellectual content might have increased the intervention’s complexity and reduced its ease of use.

### Limitations

This study’s merits include its use of a randomized controlled design with an aCG receiving concise digital sleep hygiene psychoeducation, a 6-month follow-up, and clinical interviews at 3 measurement points. However, there are several limitations to consider when interpreting the findings. First, the absence of a third nontreatment condition makes it challenging to draw conclusions about the efficacy of brief digital sleep hygiene psychoeducation, despite positive pre-to-postintervention effects. Second, the rigorous inclusion process typical of an RCT may have resulted in a sample of highly motivated students, potentially not representative of a less motivated population outside of research. Third, the overrepresentation of women in the study, though common in insomnia interventions [30], should be noted. However, it is also important to consider that women are at higher risk for sleep disturbances [9]. Fourth, the study exclusively enrolled participants with heightened insomnia symptoms (ISI ≥10), precluding inferences regarding potential effects in students with milder insomnia. Fifth, the study potentially lacked sufficient power to detect small to medium effects between iCBT-I and the aCG because the power analysis was based on a meta-analytic effect compared to waitlist controls that showed heterogeneity in sleep-related outcomes [30]. Sixth, feedback use was minimal and not systematically documented. Previous studies using the same feedback mechanism found low demand around 10%, leading to the conclusion in our study that the intervention was practically unguided [92]. Seventh, due to the varying lengths of the interventions, participants may have known which intervention was the intervention of interest and which one was the comparator. Eighth, circadian rhythm sleep disorders were neither specifically excluded nor classified, which may have broadened the target group beyond individuals with insomnia. However, insomnia diagnosis was also assessed as a secondary outcome using the Structured Clinical Interview for Sleep Disorders. This interview adheres to *DSM-5* criteria, ensuring that insomnia is not better explained by, nor does it occur exclusively in the context of, another sleep-wake disorder. Finally, sleep efficiency was measured using the PSQI instead of a sleep diary, which is widely regarded as the gold standard.

### Future Research

The study’s findings raise questions about the specific components and dose contributing to iCBT-I’s efficacy in students. Further research is required to understand the mechanisms that drive changes in sleep behavior in the short and long term resulting from psychoeducation or multicomponent iCBT-I [93]. Dismantling and component studies can help identify effective treatment components for designing resource-efficient and student-relevant iCBT-Is [94]. In this context, it may be necessary to align the intervention more closely with students’ specific needs and contextual factors, involving users in the design process [95]. Furthermore, investigating ultrabrief interventions could enhance treatment adherence among students, as they may prefer shorter, more manageable programs alongside their academic commitments. Moreover, future studies should investigate who benefits most from sleep hygiene psychoeducation versus multicomponent iCBT-I, and under what conditions. For example, students whose insomnia symptoms stem primarily from poor sleep hygiene may respond well to unguided sleep hygiene psychoeducation. In contrast, those with more complex etiologies, a chronic course of poor sleep hygiene or insomnia, more severe symptoms, or comorbid conditions such as major depression or anxiety disorders may require more comprehensive interventions. These might include additional CBT-I components, such as sleep restriction therapy, or more intensive guidance to enhance adherence to the intervention. In this respect, a cost-effectiveness analyses, including financial and personal investments in iCBT-I in relation to its long-term effect in comparison to psychoeducation should be investigated. For example, personalizing iCBT-I by tailoring guidance levels and incorporating continuous assessments on patient-relevant outcomes might optimize outcomes [96].

### Conclusions

In conclusion, this study revealed that self-help iCBT-I yielded a moderate effect on insomnia severity after 6 months compared to a single digital sleep hygiene psychoeducation session. However, by the 8-week posttreatment mark, iCBT-I was not superior in reducing insomnia severity compared to digital sleep hygiene psychoeducation. Further research is warranted to identify effective treatment components, particularly within routine care settings, to improve concise iCBT-Is for implementation in stepped-care approaches to address student insomnia.

## Appendix

Multimedia Appendix 1 Overview of outcomes: completer rates, treatment responses, and diagnostic results.

Multimedia Appendix 2 CONSORT-eHEALTH checklist (V 1.6.1).

## Abbreviations

aCG: active control group
ANCOVA: analysis of covariance
CBT-I: cognitive behavioral therapy for insomnia
CONSORT: Consolidated Standards of Reporting Trials
CONSORT-EHEALTH: Consolidated Standards of Reporting Trials of Electronic and Mobile Health Applications and Online Telehealth
DSM-5: Diagnostic and Statistical Manual of Mental Disorders
iCBT-I: internet-based cognitive behavioral therapy for insomnia
ISI: Insomnia Severity Index
ITT: intention-to-treat
MDD: major depressive disorder
NNT: number needed to treat
PSQI: Pittsburgh Sleep Quality Index
RCT: randomized controlled trial
SMD: standardized mean difference

## Data Availability

The datasets generated or analyzed during this study are available from the corresponding author on reasonable request..
