# Supplementary material for: Efficacy of a Self-Guided Internet Intervention With Optional On-Demand Feedback Versus Digital Psychoeducation on Sleep Hygiene for University Students With Insomnia: Randomized Controlled Trial
Source: J Med Internet Res. 2025 May 8;27:e58024. doi: 10.2196/58024 (PMC12099275; doi:10.2196/58024)
Supplement: Multimedia Appendix 1 [file jmir_v27i1e58024_app1.docx]

# **Appendix**

**Table S1.** Means and SDs of the iCBT-I and the aCG at T1, T2 and T3 for the study-completer sample.

| Outcome | T1 | | | | | | | T2^a^ | | | | | | |  | | | T3^a^ | |  | |  |
| --- | --- | --- | --- | --- | --- | --- | --- | --- | --- | --- | --- | --- | --- | --- | --- | --- | --- | --- | --- | --- | --- | --- |
|  | iCBT-I | | | aCG | | | | iCBT-I | | | | aCG | | | iCBT-I | | | | | aCG | | |
|  | *M* | *SD* | *M* | | | *SD* | *M* | | *SD* | | *M* | | *SD* | *M* | | | *SD* | | *M* | | *SD* | |
| **Primary Outcome** |  | | | |  | | | | |  | | | | | |  | | | | | | |
| Insomnia severity (ISI)^b^ | 16.51 | 3.65 | 16.29 | | | 3.09 | 11.03 | | 4.89 | | 12.34 | | 4.10 | 9.58 | | | 4.82 | | 12.35 | | 5.21 | |
| **Secondary Outcomes** |  | | | |  | | | | |  | | | | | |  | | | | | | |
| Sleep quality (PSQI)^c^ | 1.00 | 0.48 | 1.04 | | | 0.52 | 1.48 | | 0.68 | | 1.40 | | 0.50 | 2.24 | | | 0.61 | | 2.36 | | 0.55 | |
| Sleep efficiency (PSQI)^d^ | 73.10 | 15.28 | 75.04 | | | 12.94 | 81.72 | | 13.40 | | 78.24 | | 11.93 | 83.73 | | | 9.61 | | 80.47 | | 12.50 | |
| Cognitive irritation (IS)^e^ | 13.47 | 4.77 | 12.33 | | | 5.24 | 12.47 | | 4.86 | | 11.05 | | 5.21 | 12.00 | | | 4.91 | | 11.93 | | 5.42 | |
| Worrying (PSWQ)^f^ | 9.04 | 3.92 | 9.28 | | | 4.03 | 7.79 | | 3.66 | | 8.23 | | 3.67 | 7.97 | | | 3.88 | | 8.12 | | 4.07 | |
| Recovery experiences (REQ)^g^ | 48.31 | 6.90 | 49.93 | | | 8.12 | 49.47 | | 8.23 | | 50.95 | | 8.25 | 51.09 | | | 8.78 | | 52.55 | | 9.17 | |
| REQ_psych. detachment^h^ | 10.62 | 3.26 | 11.71 | | | 3.31 | 11.34 | | 3.16 | | 12.00 | | 3.43 | 12.30 | | | 3.28 | | 12.40 | | 3.95 | |
| REQ_relaxation | 12.71 | 2.71 | 12.18 | | | 2.83 | 13.01 | | 2.60 | | 13.18 | | 2.67 | 13.15 | | | 3.01 | | 13.23 | | 3.45 | |
| REQ_mastery | 10.31 | 3.20 | 11.20 | | | 3.75 | 10.84 | | 3.37 | | 10.80 | | 3.92 | 10.70 | | | 3.01 | | 11.88 | | 3.38 | |
| REQ_control | 14.67 | 3.03 | 14.84 | | | 2.66 | 14.24 | | 3.07 | | 14.98 | | 2.42 | 14.94 | | | 3.26 | | 15.05 | | 2.54 | |
| Recovery activities (ReaQ)^i^ | 30.02 | 10.06 | 28.11 | | | 10.06 | 30.84 | | 10.74 | | 32.14 | | 12.17 | 31.27 | | | 8.33 | | 29.16 | | 10.77 | |
| Recuperation in sleep (SFB)^j^ | 17.82 | 4.91 | 17.73 | | | 4.17 | 20.59 | | 5.78 | | 19.30 | | 5.60 | 21.15 | | | 5.47 | | 19.70 | | 4.81 | |
| Presenteeism (PSS Subscale WIS)^k^ | 46.22 | 15.70 | 48.78 | | | 13.33 | 42.43 | | 14.62 | | 43.47 | | 14.29 | 45.45 | | | 16.43 | | 43.66 | | 13.21 | |
| Procrastination (PFS)^l^ | 21.58 | 8.13 | 21.13 | | | 7.07 | 19.18 | | 6.82 | | 20.52 | | 6.86 | 20.76 | | | 7.51 | | 21.05 | | 6.57 | |

*Notes.* iCBT-I = intervention group, aCG = active control group, M = mean, SD = standard deviation. ^a^Missing data imputed by multiple imputation, ^b^ISI (0-28) higher scores reflect higher insomnia symptoms, ^c^ Sleep quality (PSQI; 0-3) lower scores reflect better sleep quality. ^d^Sleep efficiency (PSQI; 0-100) higher scores indicate increased sleep efficiency, ^e^ISK (3-21) higher scores indicate increased cognitive irritation. ^f^PSWQ (0-18) higher scores reflect higher worrying, ^g^REQ (16-80) higher scores reflect better recovery experiences, ^h^REQ psychological, detachment, relaxation, mastery, control (4-20) higher scores reflect better recovery experiences, ^i^ReaQ (0-84) higher scores indicate increased recuperation, ^j^SFB (8-40) higher items reflect higher recuperation in sleep, ^k^PSS Subscale WIS (0-100) higher scores reflect a lower level of presenteeism, ^l^PFS (7-35) higher items represent higher procrastination behavior.

**Table S2.** Means and SDs of the iCBT-I and the aCG at T1, T2 and T3 for the intervention-completer sample.

| Outcome | T1 | | | | | | T2^a^ | | | | | |  | | | T3^a^ | |  | |  |
| --- | --- | --- | --- | --- | --- | --- | --- | --- | --- | --- | --- | --- | --- | --- | --- | --- | --- | --- | --- | --- |
|  | iCBT-I | | aCG | | | iCBT-I | | | | aCG | | iCBT-I | | | | | aCG | | | |
|  | *M* | *SD* | *M* | | *SD* | *M* | | *SD* | | *M* | *SD* | *M* | | | *SD* | | *M* | | *SD* | |
| **Primary Outcome** |  | | |  | | | | |  | | | | |  | | | | | | |
| Insomnia severity (ISI)^b^ | 15.61 | 3.08 | 16.29 | | 3.09 | 10.00 | | 4.25 | | 12.36 | 4.16 | 9.00 | | | 4.90 | | 12.44 | | 5.39 | |
| **Secondary Outcomes** |  | | |  | | | | |  | | | | |  | | | | | | |
| Sleep quality (PSQI)^c^ | 1.03 | 0.48 | 1.04 | | 0.52 | 1.65 | | 0.74 | | 1.40 | 0.49 | 2.39 | | | 0.59 | | 2.36 | | 0.57 | |
| Sleep efficiency (PSQI)^d^ | 75.56 | 12.13 | 75.04 | | 12.94 | 83.44 | | 11.17 | | 78.34 | 11.96 | 83.47 | | | 8.62 | | 80.51 | | 12.87 | |
| Cognitive irritation (IS)^e^ | 14.03 | 4.61 | 12.33 | | 5.24 | 12.36 | | 5.11 | | 11.01 | 5.23 | 11.56 | | | 4.94 | | 11.88 | | 5.51 | |
| Worrying (PSWQ)^f^ | 9.13 | 3.64 | 9.51 | | 4.16 | 7.26 | | 3.24 | | 8.20 | 3.71 | 7.52 | | | 4.04 | | 8.05 | | 4.20 | |
| Recovery experiences (REQ)^g^ | 48.90 | 7.15 | 50.89 | | 8.29 | 52.64 | | 8.78 | | 52.73 | 9.48 | 52.64 | | | 8.78 | | 52.73 | | 9.48 | |
| REQ_psych. detachment^h^ | 10.42 | 3.10 | 11.71 | | 3.31 | 11.84 | | 3.02 | | 11.97 | 3.47 | 13.01 | | | 3.41 | | 12.45 | | 4.08 | |
| REQ_relaxation | 12.83 | 2.70 | 12.17 | | 2.83 | 13.63 | | 2.23 | | 13.16 | 2.71 | 13.62 | | | 2.92 | | 13.22 | | 3.60 | |
| REQ_mastery | 10.39 | 2.95 | 11.20 | | 3.75 | 10.98 | | 3.53 | | 10.80 | 3.95 | 10.48 | | | 3.24 | | 11.96 | | 3.49 | |
| REQ_control | 15.26 | 3.16 | 14.84 | | 2.66 | 15.07 | | 2.51 | | 14.95 | 2.44 | 15.67 | | | 3.14 | | 15.06 | | 2.60 | |
| Recovery activities (ReaQ)^i^ | 29.42 | 8.96 | 28.11 | | 10.06 | 33.59 | | 9.88 | | 32.19 | 12.30 | 32.18 | | | 9.00 | | 29.26 | | 11.11 | |
| Recuperation in sleep (SFB)^j^ | 19.23 | 4.21 | 17.73 | | 4.17 | 22.21 | | 5.28 | | 19.29 | 5.67 | 21.67 | | | 6.82 | | 19.82 | | 5.02 | |
| Presenteeism (PSS - WIS)^k^ | 44.03 | 15.30 | 48.78 | | 13.33 | 40.74 | | 15.58 | | 43.40 | 14.46 | 44.17 | | | 18.12 | | 43.52 | | 13.64 | |
| Procrastination (PFS)^l^ | 20.10 | 7.90 | 21.13 | | 7.07 | 18.23 | | 6.97 | | 20.61 | 6.88 | 21.34 | | | 7.94 | | 21.09 | | 6.76 | |

*Notes.* iCBT-I = intervention group, aCG = active control group, M = mean, SD = standard deviation. ^a^Missing data imputed by multiple imputation, ^b^ISI (0-28) higher scores reflect higher insomnia symptoms, ^c^ Sleep quality (PSQI; 0-3) lower scores reflect better sleep quality. ^d^Sleep efficiency (PSQI; 0-100) higher scores indicate increased sleep efficiency, ^e^ISK (3-21) higher scores indicate increased cognitive irritation. ^f^PSWQ (0-18) higher scores reflect higher worrying, ^g^REQ (16-80) higher scores reflect better recovery experiences, ^h^REQ psychological, detachment, relaxation, mastery, control (4-20) higher scores reflect better recovery experiences, ^i^ReaQ (0-84) higher scores indicate increased recuperation, ^j^SFB (8-40) higher items reflect higher recuperation in sleep, ^k^PSS Subscale WIS (0-100) higher scores reflect a lower level of presenteeism, ^l^PFS (7-35) higher items represent higher procrastination behavior.

**Table S3.** Between-group effects at T2 and T3 for the study-completer sample.

| Outcome | |  | Between-groups effect at T2 | | | | | Between-groups effect at T3 | | | | |
| --- | --- | --- | --- | --- | --- | --- | --- | --- | --- | --- | --- | --- |
|  |  | *d* | | 95%-CI | ANCOVA | | | *d* | 95%-CI | ANCOVA | | |
|  |  |  |  |  | *df* | *F* | *p* |  |  | *df* | *F* | *p* |
|  | **Primary outcome** | | | | | | |  |  |  |  |  |
| Insomnia severity (ISI) | | -0.23 | | -0.57 to 0.11 | 1, 80 | 2.26 | .14 | -0.52 | -0.96 to -0.08 | 1, 73 | 5.86 | .02 |
|  | **Secondary outcomes** | | | | | | |  |  |  |  |  |
| Sleep quality (PSQI) | | 0.02 | | -0.03 to 0.06 | 1, 75 | 0.40 | .53 | -0.27 | -0.08 to 0.02 | 1, 63 | 1.09 | .30 |
| Sleep efficiency | | 0.94 | | 0.15 to 1.71 | 1, 80 | 2.99 | .09 | 0.71 | -0.17 to 1.58 | 1, 73 | 2.07 | .16 |
| Cognitive irritation (ISK) | | 0.13 | | -0.23 to 0.49 | 1, 79 | 2.43 | .12 | 0.13 | -0.23 to 0.49 | 1, 73 | 0.00 | .95 |
| Worrying (PSWQ) | | -0.05 | | -0.36 to 0.25 | 1, 79 | 0.31 | .58 | 0.02 | -0.33 to 0.37 | 1, 73 | 0.03 | .87 |
| Recovery experiences (REQ) | | -0.10 | | -0.58 to 0.55 | 1, 79 | 1.04 | .31 | 0.06 | -0.59 to 0.70 | 1, 73 | 0.80 | .37 |
| REQ_psychological detachment | | 0.05 | | -0.19 to 0.28 | 1, 79 | 1.21 | .27 | 0.11 | -0.19 to 0.40 | 1, 73 | 0.02 | .90 |
| REQ_relaxation | | -0.06 | | -0.26 to 0.64 | 1, 79 | 0.15 | .81 | -0.05 | -0.29 to 0.19 | 1, 73 | 0.02 | .90 |
| REQ_mastery | | 0.11 | | -0.15 to 0.36 | 1, 79 | 0.01 | .94 | -0.08 | -0.31 to 0.14 | 1, 73 | 4.20 | .04 |
| REQ_control | | -0.12 | | -1.29 to 0.05 | 1, 79 | 2.93 | .09 | 0.00 | -0.20 to 0.20 | 1, 73 | 0.04 | .84 |
| Recovery activities (ReaQ) | | -0.47 | | -1.35 to 0.40 | 1, 79 | 0.33 | .57 | -0.47 | -1.34 to 0.40 | 1, 73 | 1.16 | .29 |
| Recuperation in sleep (SFB) | | 0.18 | | -0.19 to 0.58 | 1, 80 | 1.78 | .19 | 0.21 | -0.19 to 0.61 | 1, 73 | 1.97 | .16 |
| Procrastination (PFS) | | -0.30 | | -0.62 to 0.01 | 1, 79 | 2.67 | .11 | -0.07 | -0.47 to 0.32 | 1, 73 | 0.08 | .78 |
| Presenteeism (PSS) | | 0.14 | | -0.80 to 0.55 | 1, 79 | 1.09 | .67 | 0.60 | -0.56 to 1.75 | 1, 73 | 0.36 | .55 |

*Notes.* ANCOVA = analysis of covariance, CI = confidence interval, *d* = Cohen’s d, *df* = degrees of freedom, *p* = p value, T2=8-week post-treatment, T3=6-month follow-up

**Table S4.** Between-group effects at T2 and T3 for the intervention-completer sample.

| Outcome | Between-groups effect at T2 | | | | | Between-groups effect at T3 | | | | |
| --- | --- | --- | --- | --- | --- | --- | --- | --- | --- | --- |
|  | *d* | 95%-CI | ANCOVA | | | *d* | 95%-CI | ANCOVA | | |
|  |  |  | *df* | *F* | *p* |  |  | *df* | *F* | *p* |
|  | **Primary outcome** | | | | |  |  |  |  |  |
| Insomnia severity (ISI) | -0.49 | -0.95 to -0.02 | 1, 4245.70 | 5.73 | .02 | -0.61 | -1.10 to -0.13 | 1, 1795.8 | 6.92 | <.01 |
|  | **Secondary outcomes** | | | | |  |  |  |  |  |
| Sleep quality (PSQI) | 0.40 | -0.09 to 0.92 | 1, 1430.58 | 2.58 | .11 | 0.07 | -0.44 to 0.57 | 1, 3691.27 | 0.18 | .67 |
| Sleep efficiency | 0.41 | 0.01 to 0.81 | 1, 935.50 | 4.33 | .04 | 0.24 | -0.20 to 0.68 | 1, 2307.55 | 1.35 | .25 |
| Cognitive irritation (ISK) | 0.06 | -0.33 to 0.45 | 1, 10203.47 | 1.77 | .18 | -0.21 | -0.66 to 0.23 | 1, 8373.10 | 0.129 | .72 |
| Worrying (PSWQ) | -0.24 | -0.71 to 0.24 | 1, 2444.49 | 1.19 | .27 | -0.10 | -0.56 to 0.36 | 1, 7731.87 | 0.33 | .57 |
| Recovery experiences (REQ) | 0.15 | -0.25 to 0.55 | 1, 7186.28 | 0.23 | .63 | 0.07 | -0.36 to 0.49 | 1, 14482.01 | 0.11 | .74 |
| REQ_psychological detachment | 0.193 | -0.23 to 0.61 | 1, 9277.51 | 0.11 | .74 | 0.34 | -0.12 to 0.80 | 1, 1990.06 | 0.463 | .49 |
| REQ_relaxation | 0.09 | -0.36 to 0.53 | 1, 3507.38 | 0.66 | .42 | -0.00 | -0.43 to 0.42 | 1, 2138.83 | 0.31 | .58 |
| REQ_mastery | 0.18 | -0.22 to 0.58 | 1, 21229.66 | 0.10 | .75 | -0.29 | -0.69 to 0.12 | 1,2400.53 | 4.83 | .03 |
| REQ_control | -0.4 | -0.43 to 0.36 | 1, 6543.99 | 0.163 | .69 | 0.14 | -0.27 to 0.55 | 1, 2280.72 | 1.07 | .30 |
| Recovery activities (ReaQ) | 0.05 | -0.37 to 0.48 | 1, 3239.05 | 0.33 | .56 | 0.21 | -0.22 to 0.64 | 1, 1469.92 | 1.64 | .20 |
| Recuperation in sleep (SFB) | 0.32 | -0.08 to 0.73 | 1, 3575.13 | 6.78 | <.01 | 0.16 | -0.26 to 0.58 | 1, 1777.20 | 2.54 | .11 |
| Procrastination (PFS) | -0.23 | -0.52 to 0.06 | 1, 2001.49 | 5.75 | .02 | 0.13 | -0.24 to 0.49 | 1, 11192.98 | 0.11 | .75 |
| Presenteeism (PSS) | 0.03 | -0.35 to 0.42 | 1, 2755.27 | 0.84 | .36 | 0.19 | -0.24 to 0.63 | 1, 27747.55 | 0.11 | .74 |

*Notes.* ANCOVA = analysis of covariance, CI = confidence interval, *d* = Cohen’s d, *df* = degrees of freedom, *p* = p value, T2=8-week post-treatment, T3=6-month follow-up

**Table S5:** Treatment responses based on the intention-to-treat sample.

|  | **iCBT-I *(n/N), %*** | **aCG *(n/N), %*** | **Chi-Square Test *χ^2^(1)* (*N=90*)** | **Odds ratio  *(95%-CI)*** | ***NNT  (95%-CI)*** |
| --- | --- | --- | --- | --- | --- |
| **T2** |  |  |  |  |  |
| Symptom-free status | 9/45, 20.0 | 6/45, 13.3 | 0.32, *p* = .57 | 1.60 (0.52 – 5.33) | 15.00 (-11.53 – 4.54) |
| RCI improvement | 5/45, 11.1 | 7/45, 15.6 | 0.07, *p* = .76 | 0.69 (0.18–2.39) | -22.50 (-5.41 – 10.45) |
| **T3** |  |  |  |  |  |
| Symptom-free status | 19/45, 42.2 | 7/45, 15.6 | 6.54, *p* = .01 | 3.86 (1.46 – 11.29) | 3.75 (2.24 – 11.41) |
| RCI improvement | 16/45, 35.6 | 7/45, 15.6 | 3.74, *p* = .05 | 2.93 (1.09 – 8.63) | 5.00 (2.66 – 40.70) |

*Notes.* iCBT-I = intervention group, aCG = active control group, NNT = numbers needed to treat, RCI = reliable change index, 95% CI =
confidence interval, *p* = p value.

**Table S6:** Treatment responses based on the study-completer sample.

|  | **iCBT-I *(n/N), %*** | **aCG *(n/N), %*** | **Chi-Square Test *χ^2^(1)* (*N=83/76*)** | **Odds ratio  *(95%-CI)*** | ***NNT  (95%-CI)*** |
| --- | --- | --- | --- | --- | --- |
| **T2** |  |  |  |  |  |
| Symptom-free status | 7/39, 18.0 | 6/44, 13.6 | 0.06, *p* = .81 | 1.38 (0.41 – 4.79) | 23.19 (-8.75 – 4.99) |
| RCI improvement | 3/39, 7.7 | 7/44, 18.0 | 0.66, *p* = .42 | 0.46 (0.09 – 1.83) | -12.17 (-4.57 – 18.35) |
| **T3** |  |  |  |  |  |
| Symptom-free status | 13/33, 39.4 | 6/43, 14.0 | 5.16, *p* = .02 | 3.89 (1.31 – 12.82) | 3.93 (2.22 – 17.20) |
| RCI improvement | 10/33, 30.3 | 6/43, 14.0 | 2.10, *p* = .15 | 2.62 (0.84 – 8.83) | 6.12 (-40.95 – 2.85) |

*Notes.* iCBT-I = intervention group, aCG = active control group, NNT = numbers needed to treat, RCI = reliable change index, 95% CI = confidence interval, *p* = p value.

**Table S7:** Treatment responses based on the intervention-completer sample.

|  | **iCBT-I *(n/N), %*** | **aCG *(n/N), %*** | **Chi-Square Test *χ^2^(1)* (*N=76*)** | **Odds ratio  *(95%-CI)*** | ***NNT  (95%-CI)*** |
| --- | --- | --- | --- | --- | --- |
| **T2** |  |  |  |  |  |
| Symptom-free status | 7/31, 22.6 | 6/45, 13.3 | 0.55, *p* = .46 | 1.87 (0.55 – 6.63) | 10.81 (-11.75 – 3.70) |
| RCI improvement | 3/31, 9.7 | 7/45, 15.6 | 0.16, *p* = .69 | 0.60 (0.11–2.40) | -17.01 (-4.82 – 11.15) |
| **T3** |  |  |  |  |  |
| Symptom-free status | 13/31, 41.9 | 7/45, 15.6 | 5.30, *p* = .02 | 3.81 (1.32 – 11.94) | 3.79 (2.14 – 16.57) |
| RCI improvement | 10/31, 32.3 | 7/45, 15.6 | 2.07, *p* = .15 | 2.54 (0.84 – 8.09) | 5.99 (-34.88 – 2.76) |

*Notes.* iCBT-I = intervention group, aCG = active control group, NNT = numbers needed to treat, RCI = reliable change index, 95% CI = confidence interval, *p* = p value.

**Table S8.** Descriptive statistics for depression diagnosis for Study Completer

|  | **iCBT-I**  ***(n/N), %*** | **aCG**  ***(n/N), %*** |
| --- | --- | --- |
| T1 | 0/45, 0.0 | 1/45, 2.2 |
| T2 | 1/33, 3.0 | 0/29, 0.0 |
| T3 | 0/20, 0.0 | 1/30, 3.3 |

*Notes.* iCBT-I = intervention group, aCG = active control group.

**Table S9.** Logistic regressions of insomnia diagnosis on the treatment group adjusted for insomnia severity at baseline, analysis of subset who received insomnia diagnosis at baseline.

|  | **iCBT-I**  ***(n/N), %*** | **aCG**  ***(n/N), %*** | **Logistic regression**  ***χ^2^(2)* estimate, *p*** | **Odds ratio  *(95%-CI)*** | **Risk reduction  *(95%-CI)*** | ***NNT  (95%-CI)*** |
| --- | --- | --- | --- | --- | --- | --- |
| **T2** |  |  |  |  |  |  |
| Intention to treat | 21/43, 48.8 | 27/43, 62.8 | -0.64, *p* = .18 | 0.52  (0.21 – 1.34) | 0.48  (-0.39 – 0.87) | 7.36  (-5.91 – 2.86) |
| Study completer | 15/31, 48.4 | 23/27, 85.2 | -0.55, *p* = .27 | 0.58  (0.21–1.55) | 0.42  (-0.39–0.87) | 6.67  (-11.95 – 2.61) |
| Intervention completer | 13/29, 44.8 | 27/43, 62.8 | -0.74, *p* = .16 | 0.48  (0.17 – 1.36) | 0.52  (-0.39 – 0.87) | 5.46  (-44.10 – 2.38) |
| **T3** |  |  |  |  |  |  |
| Intention to treat | 16/43, 37.2 | 24/43, 55.8 | -0.86, *p* = .16 | 0.42  (0.13 – 1.39) | 0.58  (-0.39 – 0.87) | 5.02  (2.59 – 2.90) |
| Study completer | 8/25, 32.0 | 15/33, 45.5 | -1.12, *p* = .06 | 0.33  (0.10–1.03) | 0.67  (-0.39–0.87) | 4.44  (-50.42 – 2.12) |
| Intervention completer | 8/29, 27.6 | 23/43, 53.5 | -0.99, *p* = .07 | 0.48  (0.17 – 1.33) | 0.52  (-0.39 – 0.87) | 4.24  (-206.27 – 2.17) |

*Notes.* iCBT-I = intervention group, aCG = active control group, NNT = numbers needed to treat, 95%-CI = confidence interval, *p* = p value.
